# Assessment of the Genetic Diversity Among Potato Cultivars from Different Geographical Areas Using the Genomic and EST Microsatellites

**DOI:** 10.15171/ijb.1280

**Published:** 2016-12

**Authors:** Haleh Salimi, Masoud Bahar, Aghafakhr Mirlohi, Majid Talebi

**Affiliations:** Department of Biotechnology, College of Agriculture, Isfahan University of Technology, Isfahan, Iran

**Keywords:** EST-SSR, Genetic diversity, Microsatellite, Potato

## Abstract

**Background:**

Potato has a narrow genetic base which is due to its development, as it takes its genetic root from a few genotypes originated from South America.

**Objectives:**

The objective of this study was to assess the genetic relationships among potato (*Solanum tuberosum* L.) genotypes originated from different geographical regions.

**Materials and Methods:**

This study has rendered 25 useful SSRs and EST-SSRs that were located in pre-existing genetic maps, fingerprinted in a collection of the 47 potato genotypes from America, Europe and Iran.

**Results:**

The number of alleles per locus ranged from 2 to 9 with an average of 6.22 alleles per locus. UPGMA dendrogram, constructed from microsatellite data based on Jaccard similarity coefficient slightly clustered the American and European potatoes according to their geographical distribution. Iranian genotype, "Istanbuli", joined to a group with American genotype. The results indicated that American genotypes show the highest expected heterozygosity compared to the European genotype. This result was expected due to the narrow genetic base of European potatoes considering their origin from a limited number of introductions.

**Conclusions:**

It could be concluded that SSR is an appropriate marker for evaluating genetic diversity within and among potatoes from different geographical regions.

## 1. Background

Among the major food crops, potato (*Solanum tuberosum* L., Solanaceae family) is currently the subject of the highest production rate in the most developing countries. Potato is spread in most countries in Europe as an agricultural crop and consumed as a food product. The most important cause of its distribution was providing food, preservation, and eradication of the poverty (1). Potato was first introduced from Europe into America in 1621. In Iran, during the era of the Safavid dynasty potato was brought to this country by Southeast Asian-European traders. As well, Potato was brought to Iran by Sir John Malcolm, the British consular to Iran on a diplomatic mission that concedes the period of Fath Ali Shah’s ruling on Iran who ordered for the cultivation of the plant in a village called Pashand. Additionally, potato was also introduced into northern parts of Iran from Russia from which it was exported to the other parts of Russia and Iran as well as Azerbaijan. Unfortunately, there is insufficient information available regarding the crop’s subsequent distribution. However, the persevering use of local cultivars like Pashandi and Istanbuli, which are preferred for their cooking qualities, illustrates a long history of the local cultivation in the mountainous parts of northern Iran (2).

The variability in the potato geographical distribution raises the question with regards to their true origin by morphologic (color of skin and flesh of potato tubers) and genetic characteristics (such as protection against many devastating pests and pathogens). Together, these attributes present a significant barrier to the potato improvement using classical breeding approaches. Little is known about the genetic diversity of the improved *Solanum* gene pool established over the past century. Providing genome sequence of the potato is a major challenge to which breeders are faced in programs related to the potato breeding. In addition, due to its considerable value in providing food security worldwide, much effort is recently being focused on developing molecular techniques in order to facilitate management and utilization of the plant’s genetic resources (3). Identification of the potato cultivars based on phenotypic characteristics, which is hardly identifiable, time-consuming, and environmentdependent, results in a high risk of misclassification (4). Several molecular marker techniques have been utilized for different potato genetic resources. These methods have been compared to assess the most efficient method in potato germplasm identification and the evaluation of genetic diversity (5-7).

Simple sequence repeat (SSR) or microsatellite markers are highly polymorphic, abundant in the genome, and co-dominant. The first potato SSR study was based on DNA sequences from public databases (8). SSR markers have afterward been used for evaluation of the genetic diversity among *S. tuberosum* cultivars (9-10). Milbourne *et al*. (1998) have identified about 112 potato microsatellites which were located on all 12 chromosomes of the genome (11).

Subsequently, SSRs has been used for studying the genetic relationship and distances between wild and cultivated potato cultivars (12-16). SSR markers have been used successfully for characterization of the potato gene pool in several countries and SSR fingerprints have been suggested as one of the main techniques in the cultivar certification process (17-19). The recent trends in using EST-derived microsatellite markers in comparison to the genomic library derived microsatellites are driving attention of the crop scientists (20-21).

## 2. Objectives

Although Iran is definitely one of the potato producers in the Middle East, reliable information on the relationships among the cultivated potatoes in Iran, also in addition to the origin, and characteristics of an Iranian potato “Istanbuli” is limited. Furthermore, approximately all varieties which are cultivated for the commercial purposes and used in the evaluation of the breeding programs were introduced from European countries, especially from Netherlands and Germany, nd recently from the United States. Therefore, as the main goal, the focus of the present study was an investigation of the genetic diversity and phylogenetic relationships among European, American, and an Iranian (Istanbuli) potato genotypes using SSR and EST-SSR markers. In addition, there were some colorful potato tubers that have been introduced to Iran which we didn’t have any information about their phylogenetic relationship with the other potatoes that have been classified by several molecular markers (22). Therefore, we used several EST-SSRs which are linked to anthocyanin biosynthesis, the key genes that may differentiate colorful cultivars such as Purple Pelisse and Purple majesty from the white cultivars.

## 3. Materials and Methods

### 3.1. Plant Materials and DNA Extraction

A total of 47 potato genotypes originated from Europe and America were used as plant material (Table 1). The leaves were collected from *in vitro* propagated cultivars, washed three times in distilled water, were frozen in liquid nitrogen, and kept at - 80°C. Genomic DNA was extracted using the CTAB method described by Murray and Thompson, 1980 (23). The quality and quantity of the DNA samples was estimated using agarose gel electrophoresis against the known concentration of the lambda DNA fragments and further verified by spectrophotometry (Beckmann, Germany). DNA samples were diluted to 20 ng.μL^-1^ concentration and stored at -20ºC.

**Table 1:** Names, pedigree and the origin of 47 potato cultivars used

** No.**	** Variety name**	** Pedigree**	**Country of origin**
1 2 3 4 5 6 7 8 9 10 11 12 13 14 15 16 17 18 19 20 21 22 23 24 25 26 27 28 29 30 31 32 33 34 35 36 37 38 39 40 41 42 43 44 45 46 47	Agria Almera Amorosa Amred Arinda Atlantic ATTX Aula Banba Bellini Burren Caesar Concorde Cosima Courage Desiree Diamant Florida Fontane Fresco Frisia Hermes Imazca Impala Jelly Kartoffel Kennebec Labadia Lutetia Maltaje Maradona Marfona Markies Milva Mondial Nicola Piccaso Purple pelisse Purple Majesty Raja Ramos Santana Sante Sinora Yukon gold Istanbuli White Purple Majesty	Quarta x Semlo BM 77-2102 x AR 80-031-20 Arinda x Impala -------------------------- Vulcano x AR 74-78-1 Wauseon x Lenape ---------------------- (H 6747 60) Clivia x (S1 x 233 47) Hydra Slaney x Estima Mondial x Felsina Marfona x Spunta Monalisa x Ropta B 1178 SVP Y66 13 636 x M 69864 (Sabina x Voran) x (MPI 41 969 377 x Flava) Lady Rosetta x HZ 81 h 202 Urgenta x Depesche Mutant of Cardinal Agria x VK69 491 Agria x AR 76-034-03 Cebeco 60- 15- 28 x Provita ZPC 69 C160 x SVP AM 66 42 DDR 5158 x SW 163/55 ---------------------------- BM 52- 72 x Biranco Marabel x 173/87/4476 ------------------------------ USDA B127 x USDA 96 56 Mondial x Van Gogh Saskia x Renska ------------------------ Cardinal x 70-66 Primura x (Craigs Bounty x Profijt) Fianna x Agria Nena x Dunja Spunta x SVP VE 66 295 Clivia x 6430 1011 Cara x Ausonia ---------------- ------------------- Elvira x CB 70 162 23 Agria x VK 69-491 Spunta x VK 69 491 SVP Y 66 13 636 x SVP AM 66 42 Agria x AM 70-2166 Norgleam x W 5279 4 -------------------------- --------------------------	Germany Netherlands Netherlands -------------- Netherlands United States United states Germany Ireland Netherlands Ireland Netherlands Netherlands Germany Europe Netherlands Netherlands Netherlands Netherlands Netherlands Netherlands Austria ------------- Netherlands Germany Germany United States Europe Netherlands Europe Netherlands Netherlands Netherlands Canada Germany- Netherlands Germany Netherlands United States United States Netherlands Europe Netherlands Netherlands Europe Canada Iran United states

### 3.2. Microsatellite Amplification

Forty-seven potato genotypes were analyzed using 25 microsatellite primer pairs (Table 2). Six ESTderived primers were designed using motif finder software (http://www.broadinstitute.org/igv/motif_finder) with the length of 18 to 20 oligonucleotides, Tm between 50 and 60°C and amplification product length between 100 -250 bp.

**Table 2:** SSRs and EST-SSRs primer sequences, repeat types, allele size ranges, and annealing temperatures

**SSR name**	**Repeat Motif**	**Primer sequences (5’-3’ Forward-Reverse)**	**Annealing temperature (°C)**	**Expected size (bp)**	** Reference **
STI001	(AAT)_n_	CAGCAAAATCAGAACCCGAT GGATCATCAAATTCACCGCT TATGTTCCACGCCATTTCAG ACGGAAACTCATCGTGCATT	54-60	188	(20)
STI007	(GTT)_n_(GAT)_n_	TGAGGGTTTTCAGAAAGGGA CATCCTTGCAACAACCTCCT	54-60	134	(20)
STI033	(AGG)_n_	CATACGCACGCACGTACAC TTCAACCTATCATTTTGTGAGTCG	64	134	(20)
STM0031	(AC)_5_ ... (AC)_3_ (GCAC) (AC)_2_ (GCAC)_2_	TGATTCTCTTGCCTACTGTAATCG CAAAGTGGTGTGAAGCTGTGA	57	155-205	(11)
STM1104	(TCT)_5_	GGGACATCACAGTCT GGTGCTCCTATTGGTG	57	164-185	(11)
POT83/84	(GT)_9_	GCGTCAGCGATTTCAGTACTA TTCAGTCAACTCCTGTTGCG	46-50	153	(12)
STM2022	(CAA)_3_...(CAA)_3_	GTGATTGGCAATCAGATTGAAA GTGTGTGGACTGTGGAGTGG	53	184-244	(11)
ST21/22	(AT)_11_	TGTGTTTGTTTTTCTGTAT AATTCTATCCTCATCTCTA	60	200	(12)
STM1031	(AT)_13_	CAACTCAAACCAGAAGGCAAA GAGAAATGGGCACAAAAAACA	55	265-325	(11)
STM3012	(CT)_4_, (CT)_8_	AACATTACAACACATTAGCA AACTTATCTGAAACTCTCGT	57	168-213	(11)
POT47/48	(TG)_10_(AG)_10_	TCAGACCGGGTTCGATGG CGGCTTGAATCATTGCCCA	47-50	204	(12)
STI053	(AT)_imp_	AATTCATGTTTGCGGTACGTC ATGCAGAAAGATGTCAAAATTGA	54-60	160	(20)
ST15/16	(AAG)_7_	GGAAGGACAGCAAACGATGT AATCGCGATGCTCTTATGCT	59	250	(12)
MYB1	(TCC)_10_	CCTACATCCCATCATAATCACA CATTCATCAAAGCCTACTCACC	60	152	This study
F3H2	(AGA)_5_	TTCCAGGAAGGACAGCAAAT CGTTTGAGAAGTTCCGAGGT	60	188	This study
MYB2	(CCT)_7_ CGC	GAAGCGACTTCCAAAATCAGA AAAGGGAGGAATAGAAACCAAAA	60	186	This study
STI012	(ATT)_n_	TCCCTGTTGCCTTGAACAAT TGGGAAAAGGTACAAAGACGA	52-58	183	(20)
STI019	(ATCT)_imp_	CTACCAGTTTGTTGATTGTGGTG	60	126	(20)
STM1049	(ATA)_6_	AGGGACTTTAATTTGTTGGACG	57	184-254	(11)

PCR amplification was performed in a total volume of 15 μL reaction mix containing 1X PCR buffer (50 mM KCl; 10 mM Tris-HCl, pH 8.3), 0.2 mM dNTPs, 0.3 μM of each forward and reverse primers, 1.5 mM MgCL2 1 unit of *Taq* DNA polymerase (CinnaGen Co.) and approximately 10-20 ng of genomic DNA. All reactions were performed in a Mastercycler gradient 96 thermocycler (Eppendorf, Hamburg, Germany). PCR cycling conditions consisted of an initial denaturation step at 95°C for 3 min, followed by 30 cycles of 95°C denaturation for 1 min, 30 s at optimal annealing temperature (Table 2) and 72°C for 1 min, followed by a final extension at 72°C for 10 minutes.

PCR products were discriminated on 12% non-denaturing polyacrylamide gel electrophoresis in 1X TBE buffer along with 100bp DNA ladder (Fermentase) and visualized by silver staining (24).

### 3.3. Data Analysis

Reproducible and clear bands were scored as binary characters (i.e., their presence (1) or absence (0)). The PowerMarker software Ver. 3.25 (25) was used to estimate the number of alleles per locus, observed heterozygosity (Ho), gene diversity (expected heterozygosity, He), and the polymorphism information content (PIC). The power of discrimination was calculated using the formula: PD = 1-Σgi^2^, where gi is the frequency of the *i*th genotype (26). Dissimilarity matrices (1000 bootstraps) were calculated for the single data based on presence/absence of the alleles using the Jaccard coefficient, and the cluster analysis was performed using unweighted paired group method with arithmetic average (UPGMA) as implemented in DARwin 5 software (27). Genetic relationships among genotypes were further analyzed by the principal component analysis (PCA) of a similarity matrix according to the extracted Eigen vectors in NTSYSpc version 2.02i.

POPGENE 1.32 software (28) was used to calculate the effective number of alleles per locus (*Ne*), expected heterozygosity (He), Shannon’s Information index (*I*), and the gene flow (*Nm*).

The program STRUCTURE 2.3.3 was applied to classify individuals into their origin and identification of the genetic relationship as well as ancestral source populations of the potato’s genotypes (29; available at http://pritch.bsd.uchicago.edu/structure.html) by two independent runs of K = 1-2 using the admixture model with 10,000 repetitions of MCMC.

## 4. Results

### 4.1. Microsatellite Amplification and Allelic Variation

All the 25 SSR and EST-SSR primers resulted in amplification of fragment with expected size range properly. Due to the tetraploid nature of the assayed genotypes, one to four different alleles per genotype were expected. Estimating the Hardy-Weinberg equilibrium (HWE) of the polymorphic loci revealed that most loci, except STI033, STM0031, STM2022, ST21/22, F_3_H_2_, and STI019 have significantly deviated from HWE (P < 0.01) (Table 3). Considerable deviation from HWE is probably due to vegetative propagation of the *Solanum tuberosum*.

The number of alleles detected per SSR locus ranged from 2 (STM1049) to 9 (STM1104), respectively (Table 3). A total of 150 alleles were identified with an average of 2.96 alleles per locus, which 145 alleles was polymorphic.

**Table 3:** The summary of statistical analysis regarding genetic diversity across all potato accessions based on 20 microsatellite loci

**Marker name**	**Allele number**	**He**	**Ho**	**PIC**	**P value**
pSTI001 STI007 STI033 STM0031 STM1104 POT83/84 STM2022 ST21/22 STM1031 STM3012 POT47/48 STI053 ST15/16 MYB1 F3H2 MYB2 STI012 STI019 STM1049 Means	4 4 5 4 9 5 4 4 7 6 4 4 3 5 3 3 4 5 2 6.22	0.65 0.55 0.56 0.52 0.54 0.74 0.27 0.25 0.51 0.56 0.50 0.57 0.24 0.54 0.48 0.35 0.33 0.61 0.30	0.43 0.25 0.57 0.41 0.07 0.52 0.09 0.21 0.52 0.86 0.46 0.66 0.00 0.54 0.52 0.21 0.43 0.44 0.00	0.59 0.50 0.49 0.44 0.52 0.70 0.26 0.23 0.42 0.60 0.46 0.50 0.22 0.51 0.39 0.30 0.30 0.54 0.34 0.42	0.0000 0.0000 0.0280 0.0290 0.0000 0.0000 1.0000 0.1200 0.0000 0.0000 0.0000 0.0000 0.0000 0.0000 0.0020 0.0000 0.0000 0.1100 0.0000

PIC: Polymorphism Information Content H_0_: Observed heterozygosity H_e_: Expected heterozygosity

PIC values for 25 microsatellites and EST markers varied from 0.12 to 0.70. The high number of polymorphic bands, considerable discriminatory power, and reliable pattern of productivity recommended SSRs as reliable tools in evaluating genetic diversity among various potato variants. In this research, the amplification efficiency of EST-SSR markers was much higher than that of genomic SSRs.

### 4.2. Cluster Analysis

The genetic relationships among 47 potato genotypes were investigated by cluster analysis using Jaccard’s similarity coefficients and UPGMA algorithm (cophenetic correlation coefficient of 0.821).

**Figure 1:**
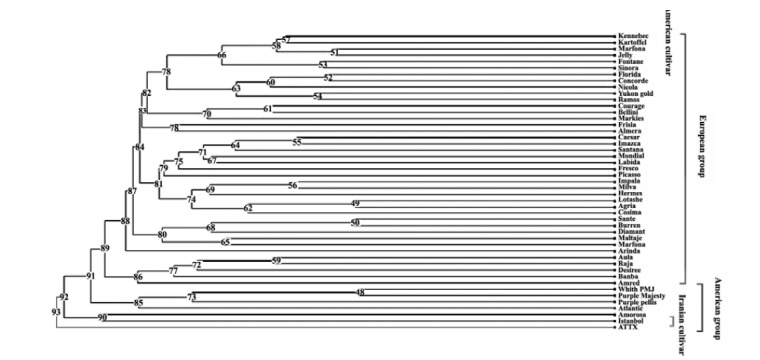
UPGMA dendrogram constructed for 47 potato genotypes studied in the present report using microsatellite markers based on the Jaccard’s coefficient. The codes refer to the abbreviations of the genotypes as shown in Table 1

UPGMA dendrogram was clustered for American and European potatoes according to their geographical origin (Figure 1). Although the European and American genotypes were not clearly discriminated, closely related American and European potatoes were classified based on their morphological characteristics such as the color of tuber skin (Data not shown). Among the studied varieties, Purple Pelisse and Purple Majesty have purple tuber peel as they classified near each other in UPGMA dendrogram. The second point is about an Iranian genotype ‘Istanbuli’ that was classified near American ones. Although some genotypes are native to America, but the introduction of these samples was attained from European collections to Iran. As indicated in the dendrogram ‘Istanbuli’ tends to group with American cultivars.

Principle component analysis (PCA) based on genetic similarity matrix has revealed that the first three principle components (PCs) account for 30.14% of the total molecular variation, showing SSR and EST-SSR markers are distributed through the genome.

Therefore, genetic relationships assessment among accessions should be established based on cluster analysis or more numbers of PCs (30).

### 4.3. Population Genetic Structure

Forty-seven potato genotypes were grouped into two groups; European and American. We waived the Iranian sample, Istanbuli, in this grouping since it was merely one genotype. All the used loci were polymorphic within and among each geographical group. The mean value of expected heterozygosity (*He*) and Shannon’s information index (*I*), as the two useful intra-region gene diversity indices, ranged from 0.4710 to 0.4879 and from 0.8306 to 0.7404, respectively. Among the groups, the highest expected heterozygosity was observed in American group.

**Figure 2:**
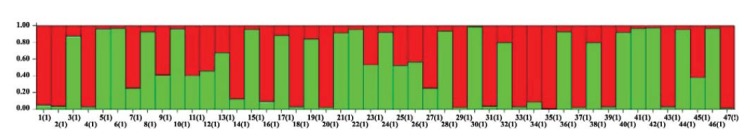
The estimated genetic structure for K= 2 obtained with the STRUCTURE program for 47 potato genotypes based on polymorphism in SSR and EST-SSR markers. Each vertical bar represents a genotype listed in Table 1 and its assignment proportion into 1 of 2 (red and green) population clusters (K)

Bayesian clustering of the information from the SSRs loci using the program: STRUCTURE has revealed that the model with K=2 explains the data satisfactorily, which suggests that the most probable number of population was two based on our present data (Figure 2). Red and green color vertical bars represent the genotypes and its assignment proportion which probably originated from America and Europe, respectively.

## 5. Discussion

The results of our research indicate that EST-SSR and SSR markers are efficient in evaluating genetic diversity and potato germplasm characterization among the different geographical regions. Our results are in agreement with the results of Milbourn *et al*. (1998) (11), Ashkenazi *et al*. (2001) (12), Ghislain *et al*. (2004) (14), and Feingold *et al*. (2005) (20). SSRs due to their simplicity, informativeness, and reproducibility are frequently nominated for practical applications such as germplasm conservation, management, and evaluation trials in order to bank various repositories (19,31-32). Besides, advancement of microsatellites utilization in plant genomes through EST-derived markers has become as a prevailing procedure (33-34). The frequency of SSRs in SSR containing ESTs can accurately reflect the density of SSRs in the transcribed region of the genome. However, because of the inadequate variability in the conserved regions of genes, many of the EST-derived markers have not been recognized as functional markers. Despite the probable advantages or disadvantages of the different methods which were used in potato fingerprinting, each of these methods has been found useful for the development of the markers in plants (5). The higher PCR amplification efficiency of EST-SSRs may be attributed to the available sequences data for primers design as there were designed from regions with the highly conserved transcribed regions, not randomly from the total genomic libraries. Therefore, due to the fact that ESTSSRs were from the highly conserved transcribed regions, they were reported to be less polymorphic but have higher transferability and a better applicability than genomic SSR markers in crops (20).

The polymorphic information content (PIC) and the polymorphism rate (P) were used to estimate the genetic diversity. High polymorphism results were observed in the primer pairs with PIC > 0.5. The mean PIC value obtained in this study was 0.42, indicating that SSR markers could discriminate medium loci polymorphism which is useful for genetic variation of the potato genotypes in this research as well as being in agreement with the results of Feingold *et al*. (2005) (20) and Ghislain *et al*. (2009) (16).

Clustering the American and European potatoes according to their geographical distribution could show the relatedness of their genetic background due to their origin and geographical distribution. These results are in agreement with the results of Bornet *et al*. (2002) (35) that have successfully classified potatoes from Argentina and Europe in two specific groups according to their geographical distribution pattern. As well, the results are in contradiction with the results reported by Esfahani *et al*. (2009) (36) that have shown a low discrimination between European and North American potatoes.

The lack of clear discrimination for Iranian genotype from that of American in the present cluster analysis reflects low genetic differentiation between them. This result is in controversy with the study which showed a genetic similarity of the Iranian genotype, Istanbuli, with the European potatoes (22). Some potatoes are native to Americans, but the introduction of these samples was attained from European collections to Iran.

The high relative gene flow (*Nm* = 3.8668) among American and European potatoes may be explained in part by the clonal propagation and existence of a common ancestor (37). The genetic diversity of the European potatoes from a limited number of introductions which could be explained to be due to a narrower genetic base (i.e. lower genetic diversity) of their origin (38) when it is compared to that of American potatoes. In general, the higher genetic diversity seems to be completely sensible due to the major region of origin. Inconsiderably, the lower genetic distances, and the higher genetic diversity among American potatoes emulate the potential for a larger number of wild and cultivated potatoes from America compared to Europe. Research have also illustrated that the gene pool of European potatoes are somehow homogenous and show a lack of variability which might be caused by the lower number of their arisen cultivars (14-15).

In conclusion, the SSR and EST-SSR markers with sufficient polymorphism can be successfully used for assessment of genetic diversity and population structure of the potato germplasm. The close genetic relationship between American and European potatoes could show the existence of common ancestors that might be due to an inherent narrow genetic base from which the potato gene pool was domesticated, combined with the historical migration of germplasm, and potato’s propagation manner.
